# Left ventricular ejection fraction and cardiac biomarkers for dynamic prediction of cardiotoxicity in early breast cancer

**DOI:** 10.3389/fcvm.2022.933428

**Published:** 2022-08-16

**Authors:** Florian Posch, Tobias Niedrist, Theresa Glantschnig, Saskia Firla, Florian Moik, Ewald Kolesnik, Markus Wallner, Nicolas Verheyen, Philipp J. Jost, Andreas Zirlik, Martin Pichler, Marija Balic, Peter P. Rainer

**Affiliations:** ^1^Division of Haematology, Department of Internal Medicine, Medical University of Graz, Graz, Austria; ^2^Clinical Institute of Medical and Chemical Laboratory Diagnostics, Medical University of Graz, Graz, Austria; ^3^Division of Cardiology, University Heart Center, Department of Internal Medicine, Medical University of Graz, Graz, Austria; ^4^Department of Cardiology, Rhythmology, and Intensive Care Medicine, KRH Klinikum Siloah, Klinikum Region Hannover GmbH, Hanover, Germany; ^5^Division of Oncology, Department of Internal Medicine, Medical University of Graz, Graz, Austria; ^6^Department of Medicine III, Klinikum rechts der Isar, TUM School of Medicine, Technical University of Munich, Munich, Germany; ^7^Department of Experimental Therapeutics, MD Anderson Cancer Center, Houston, TX, United States; ^8^BioTechMed Graz, Graz, Austria

**Keywords:** cardiotoxicity, breast cancer, trastuzumab, risk assessment, left ventricular ejection fraction, cardiac biomarkers

## Abstract

**Background/Purpose:**

This study aims to quantify the utility of monitoring LVEF, hs-cTnT, and NT-proBNP for dynamic cardiotoxicity risk assessment in women with HER2+ early breast cancer undergoing neoadjuvant/adjuvant trastuzumab-based therapy.

**Materials and methods:**

We used joint models of longitudinal and time-to-event data to analyze 1,136 echocardiography reports and 326 hs-cTnT and NT-proBNP measurements from 185 women. Cardiotoxicity was defined as a 10% decline in LVEF below 50% and/or clinically overt heart failure.

**Results:**

Median pre-treatment LVEF was 64%, and 19 patients (10%) experienced cardiotoxicity (asymptomatic *n* = 12, during treatment *n* = 19). The pre-treatment LVEF strongly predicted for cardiotoxicity (subdistribution hazard ratio per 5% increase in pre-treatment LVEF = 0.68, 95%CI: 0.48–0.95, *p* = 0.026). In contrast, pre-treatment hs-cTnT and NT-proBNP were not consistently associated with cardiotoxicity. During treatment, the longitudinal LVEF trajectory dynamically identified women at high risk of developing cardiotoxicity (hazard ratio per 5% LVEF increase at any time of follow-up = 0.36, 95% CI: 0.2–0.65, *p* = 0.005). Thirty-four patients (18%) developed an LVEF decline ≥ 5% from pre-treatment to first follow-up (“early LVEF decline”). One-year cardiotoxicity risk was 6.8% in those without early LVEF decline and pre-treatment LVEF ≥ 60% (*n* = 117), 15.9% in those with early LVEF decline or pre-treatment LVEF < 60% (*n* = 65), and 66.7% in those with early LVEF decline and pre-treatment LVEF < 60% (*n* = 3), (Gray’s test *p* < 0.0001).

**Conclusion:**

Cardiotoxicity risk is low in two thirds of women with HER2+ early breast cancer who have pre-treatment LVEF ≥ 60% and no early LVEF decline > 5% during trastuzumab-based therapy. The longitudinal LVEF trajectory but not hs-cTnT or NT-proBNP allows for a dynamic assessment of cardiotoxicity risk in this setting.

## Introduction

Cardiotoxicity is an increasingly recognized complication of antineoplastic therapy that leads to morbidity, cancer treatment discontinuation, and potentially worse long-term outcome (1). Patients with human epidermal growth factor receptor 2-positive early breast cancer (HER2+ eBC) are particularly at risk for cardiotoxicity, because modern treatment schedules for this tumor rely on two antineoplastic agent classes of significant cardiotoxic potential, namely, anthracyclines and HER2-targeted therapies (2, 3). In clinical practice, routine monitoring of left ventricular ejection fraction (LVEF) during the course of treatment is a widely adopted strategy to survey this population for cardiotoxic treatment effects. However, whether LVEF as a single measurement in time or as a trajectory over time is truly sufficient to identify patients at high risk for cardiotoxicity is currently debated (1). In addition, recent studies demonstrate that biomarkers of cardiac injury, including cardiac troponins I (cTnI), cardiac troponin T (cTnT), and N-terminal pro-brain natriuretic peptide (NT-proBNP), are correlated with LVEF measurements in patients with cancer and may predict cardiotoxic complications (4, 5).

While it is natural for physicians to re-assess prognosis and clinical management based on emerging imaging and laboratory data, quantifying the relationship between a longitudinal string of measurements and a clinical outcome is not straight-forward from a technical perspective. So-called joint models of longitudinal and time-to-event data were developed to address this issue (6). These models lend themselves both to estimating an association between a longitudinal biomarker trajectory and a clinical outcome, as well as to outcome risk predictions for individual patients conditional on their previous longitudinal biomarker trajectory (7). In this study on a large single-center population of patients with HER2+ eBC, we applied joint models of longitudinal and time-to-event data to quantify the prognostic association between longitudinal LVEF measurements and cardiotoxicity, and assessed whether LVEF trajectory can be used for dynamically predicting individual cardiotoxicity risk. Moreover, we investigated related longitudinal changes in the cardiac biomarkers high-sensitive cTnT (hs-cTnT) and NT-proBNP, and their prognostic potential in predicting cardiotoxicity independently and when added to LVEF.

## Materials and methods

### Study cohort, design, and echocardiography measurements

In this retrospective cohort study, we included all patients with HER2+ eBC who initiated neoadjuvant and/or adjuvant HER2-directed antineoplastic therapy (trastuzumab ± pertuzumab ± chemoendocrine therapy) in a curative intent between February 2006 and January 2016 in a single academic center in Europe (Division of Oncology, Medical University of Graz, Graz, Austria) and had a baseline echocardiography report and at least one follow-up echocardiography report available. Thus, *n* = 19 women without an available pre-treatment echocardiography report and *n* = 2 women without any on-treatment echocardiography reports (*n* = 2) were excluded from the analysis. As part of established care pathways in this center, echocardiography is performed every 3 months during treatment and every 6–12 months thereafter. LVEF was measured with Teichholz, Simpson, and 3D methods according to the preference of the echocardiography examiner. Echocardiography data, demographic data, tumor data, and outcome data were extracted from the hospital information system (HIS) MEDOCs as previously described, as well as from the echocardiography documentation systems at the local Division of Cardiology. Cardiotoxicity was defined as a 10% decline in LVEF below 50% and/or overt clinical heart failure as assessed by treating physicians, and could be either symptomatic or asymptomatic (8). Time-to-cardiotoxicity was defined as the time from first administration of HER2-directed treatment to the occurrence of cardiotoxicity or censoring alive on the last echocardiography date, whichever came first. The conduct of the study was approved by the local institutional review board before any data collection took place (ethics committee of the Medical University of Graz, ethikkommission@medunigraz.at, vote number: EK-Nr. 31-287 ex 18/19).

### Laboratory measurements

As part of the structured biobanking program of our institution, one 7-ml vial of whole blood was drawn into an appropriate sampling tube (VACUETTE^®^ CAT Serum Sep Clot Activator, Greiner Bio-One International GmbH) every 3–6 months in routinely indicated blood collections. The biobanking samples were further processed to gain serum, which was then aliquoted and deep-frozen at –80°C. We only included samples from patients who gave written informed consent. We quantified the concentrations of NT-proBNP and hs-cTnT on a cobas^®^ 8000 e801 analyzer (Roche Diagnostics, Mannheim, Germany) using automated electrochemiluminescence immunoassays (ECLIA) from the same manufacturer.

### Statistical methods

All the statistical analyzes were performed by FP using Stata 15.0 (Stata Corp., Houston, TX, United States). Continuous variables were reported as medians [25th–75th percentile], whereas count data were summarized as absolute frequencies (%). Medians [25th–75*^th^* percentile] instead of means ± standard deviations were chosen as measures of central tendency, because many of our examined continuous variables had skewed distributions. The distribution of data between patients with and without cardiotoxicity was evaluated by rank-sum tests, χ^2^-tests, and Fisher’s exact tests, as appropriate, whereas Spearman’s ρ was used for univariate correlation analyzes. Median follow-up was estimated with the reverse Kaplan-Meier estimator (9). Simulation of LVEF values in% from uniform distributions was used to handle discrete LVEF readouts (Supplementary Paragraph 1). The cumulative incidence of cardiotoxicity was computed with competing risk cumulative incidence estimators, treating death from any cause as the competing event of interest. Follow-up time in all the cardiotoxicity risk analyzes was truncated at 1 year after the first dose of HER2-targeted therapy. Distant recurrence-free survival was obtained with a Kaplan-Meier estimator. Cumulative incidences between two or more groups were compared by Gray’s test (10). Uni- and multivariable modeling of cardiotoxicity subdistribution hazards was performed with Fine and Gray competing risk regression models (11). Changes in LVEF and cardiac biomarkers over time were analyzed with linear mixed models with up to a cubic specification for follow-up time, a random intercept for each patient, and a random effect for linear follow-up time (12). To quantify the association between trajectories of LVEF and cardiac biomarkers with subsequent risk of cardiotoxicity, we fitted joint models of longitudinal and time-to-event data (6). Briefly, joint models estimate the association between a longitudinal process (in our case continuous longitudinal measurements of LVEF or cardiac biomarkers) and a time-to-event process (in our case the rate of cardiotoxicity). This association is represented by the association parameter α. As previously described (7), we used a linear mixed model for LVEF and cardiac biomarkers, a Weibull model for cardiotoxicity rates, and examined two specifications of α, namely, the “current parameter” specification (to be interpreted as the association between a biomarker at any time of follow-up and cardiotoxicity risk), and the “1st derivate” specification (to be interpreted as the association between the rate of change in a biomarker and cardiotoxicity risk). Missing LVEF and cardiac biomarker readings were allowed in both linear mixed models and joint models. Personalized predictions of cardiotoxicity risk conditional on LVEF and biomarker trajectories were obtained from joint models based on the dynamic prediction approach of Rizopoulos (13). The full analysis code is available upon request to FP.

## Results

### Study cohort and cardiotoxicity event rates

A total of one-hundred-eighty-five women with HER2+ eBC were included in the analysis (Table 1). Briefly, the median age of the cohort was 55 years [25th–75th percentile: 49–65], 61 (34%) patients had node-positive disease, and 103 (56%) were treated in the neoadjuvant setting. Median echocardiographic follow-up was 1.1 years, with 75 and 25% of the study cohort being followed-up with echocardiography for at least 0.9 and 1.7 years, respectively. During this interval, 19 patients (10%) experienced cardiotoxicity. All 19 cardiotoxicity events occurred during HER2-targeted treatment. However, only one-thirds of these events was symptomatic (*n* = 7, 37%), and most of the patients required modification/termination of their HER2-targeted cancer treatment (*n* = 14, 74%). In detail, the HER2-targeted treatment was not changed in n = 5 women with cardiotoxicity (26%), modified in *n* = 2 women with cardiotoxicity (11%), and permanently terminated in *n* = 12 women with cardiotoxicity (63%). Median time to cardiotoxicity was 6.7 months (25th–75th percentile: 3.4–10.2), and median maximum LVEF decline in patients with cardiotoxicity was 18% (25th–75th percentile: 14–22, range: 10–35). Overall, the 19 events corresponded to 3-, 6-, and 12-month cardiotoxicity risks of 2% (95% CI: 1–6), 4% (2–9), and 11% (7–17), respectively (Supplementary Figure 1). During an oncologic median follow-up interval of 4.5 years, 22 patients developed distant metastasis, and 9 patients died. This corresponded to a 5-year distant-recurrence-free survival (dRFS) experience of 85% (95% CI: 78–90, Supplementary Figure 2). Relatively few missing data were present (Table 1), except for baseline hs-cTnT and baseline NT-proBNP, which were both only available in 70 patients (62% missing). Among baseline variables other than LVEF and cardiac biomarkers, higher BMI but not radiotherapy for left-sided breast cancer was associated with higher cardiotoxicity risk (Table 1).

**TABLE 1:** Baseline characteristics of the study population (*n* = 185).

Variable	*n* (% miss.)	Overall (*n* = 185)	No cardiotoxicity during F/U (*n* = 166)	Cardiotoxicity during F/U (*n* = 19)	*P*
**Demographics**					
Age (years)	185 (0%)	55 [49–65]	54 [48–64]	62 [54–69]	0.066
Female sex	185 (0%)	185 (100%)	166 (100%)	19 (100%)	N/A
Body Mass Index (kg/m^2^)	140 (24%)	25 [22–30]	25 [22–29]	27 [26–31]	0.020
**Tumor and treatment characteristics**					
HER2 positive	185 (0%)	185 (100%)	166 (100%)	19 (100%)	N/A
Estrogen receptor positivity	184 (< 1%)	124 (67%)	112 (68%)	12 (63%)	0.68
Progesteron receptor positivity	184 (< 1%)	109 (59%)	99 (60%)	10 (53%)	0.54
Ki-67 (%)	142 (23%)	35 [23–45]	35 [20–45]	36 [28–53]	0.51
Tumor grade G3	179 (3%)	118 (66%)	104 (64%)	14 (82%)	0.13
TNM cT3-4	181 (2%)	12 (7%)	11 (7%)	1 (6%)	0.99
TNM cN +	180 (3%)	61 (34%)	52 (32%)	9 (50%)	0.13
TNM cM0	185 (0%)	185 (100%)	166 (100%)	19 (100%)	N/A
Left-sided breast cancer	185 (0%)	95 (51%)	84 (51%)	11 (55%)	0.730
Neoadjuvant therapy	185 (0%)	103 (56%)	92 (55%)	11 (58%)	0.84
Adjuvant radiotherapy	185 (0%)	144 (78%)	129 (78%)	15 (79%)	0.90
**Selected comorbidities**					
Coronary artery disease	185 (0%)	3 (2%)	3 (2%)	0 (0%)	0.999
Arterial hypertension	185 (0%)	55 (30%)	46 (27%)	9 (47%)	0.076
Diabetes mellitus	185 (0%)	9 (5%)	8 (5%)	1 (5%)	0.932
**Study variables**					
Baseline LVEF (%)	185 (0%)	64 [60–68]	65 [60–70]	62 [57–63]	0.016
Baseline hs-cTnT (pg/mL)	70 (62%)	5 [2–8]	5 [2–8]	9 [2–17]	0.34
Baseline NT-proBNP (pg/mL)	70 (62%)	94 [59–191]	94 [59–191]	151 [33–447]	0.69

Data are medians [25th–75th percentile] for continuous variables, and absolute frequencies (%) for count data. n (%miss.) reports the number of patients with an observed record for the respective variable (% missing). F/U, follow-up; N/A, not applicable; HER2, human epidermal growth factor receptor 2; Ki-67, proliferation index Ki-67; TNM, tumor node metastasis classification; cT, clinical tumor size according to TNM system; cN +, clinically positive nodes according to TNM system; cM0, no clinical indication of metastasis according to TNM system; LVEF, left ventricular ejection fraction; hs-cTnT, high-sensitivity cardiac troponin T; NT-proBNP, N-terminal pro-brain-natriuretic peptide.

### Pre-treatment LVEF, hs-cTnT, and NT-proBNP for cardiotoxicity risk prediction

**FIGURE 1:**
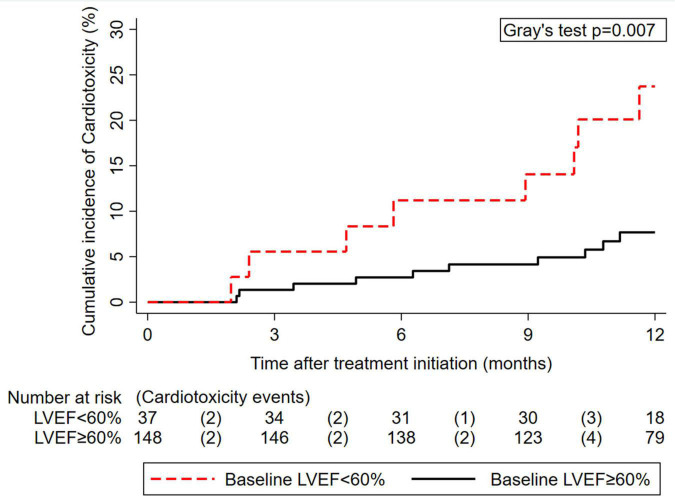
Cumulative 12-month incidence of cardiotoxicity according to pre-treatment left ventricular ejection fraction (LVEF) (*n* = 185). The numbers below the x-axis represent a risk table, with the number of patients at risk for cardiotoxicity at the beginning of each interval and the number of patients who developed cardiotoxicity during the pertinent interval in round brackets.

At baseline, median pre-treatment LVEF was 64% [60–68], and median pre-treatment levels of hs-cTnT and NT-proBNP were 5 [2–8] and 94 pg/ml [59–191], respectively (Table 1). Baseline hs-cTnT and NT-proBNP were moderately positively correlated with each other (Spearman’s ρ = 0.32, *p* < 0.001); baseline LVEF was only minimally correlated with hs-cTnT (ρ = -0.1, *p* = 0.042) but is not correlated with NT-proBNP (ρ = -0.03, *p* = 0.61). A lower pre-treatment LVEF predicted for increased cardiotoxicity risk. In detail, 12-month cardiotoxicity risk was 8% (95% CI: 4–14) in 37 patients with an LVEF < 60%, and 24% (13–42) in 148 patients with an LVEF ≥ 60% (Gray’s test *p* = 0.007, Figure 1), which corresponded to a 3.3-fold relative increase in the subdistribution hazard of cardiotoxicity for LVEF < 60% (Table 2). In the subset of 70 patients with observed pre-treatment cardiac biomarkers, patients with hs-cTnT levels > the 75*^th^* percentile of its distribution (*n* = 14) experienced a 12-month cardiotoxicity risk of 19%, whereas the corresponding estimate for those patients with ≤ this cut-off (*n* = 56) was 2% (Gray’s test *p* = 0.043, cut-off at > 8 pg/ml, Supplementary Figure 3A). The results were even more pronounced when using a cut-off of 14 pg/ml, although subgroup sizes were small (Supplementary Figure 3B). No association between hs-cTnT and cardiotoxicity risk was observed when modeling the variable as a continuous parameter in a time-to-event model (Table 2). Otherwise, cardiotoxicity risks were not statistically significantly different between patients with NT-proBNP levels ≤ (*n* = 48) and > (*n* = 22) the established upper limit of normal (ULN) at 150 pg/ml (12-month risks: 3% vs. 11%, Gray’s test *p* = 0.23, Supplementary Figure 4 and Table 2). Higher age and higher BMI were numerically but not statistically significantly associated with higher cardiotoxicity (Table 1).

**TABLE 2:** Associations between baseline and longitudinal left ventricular ejection fractions, high-sensitivity troponin T, N-terminal pro-brain natriuretic peptide, and cardiotoxicity.

Variable	(S) HR	95%CI	*P*
**Baseline variables**			
Baseline LVEF (per 5% increase)	0.68	0.48–0.95	0.026
Baseline LVEF < 60%	3.31	1.31–8.40	0.012
Baseline hs-cTnT (per 5 pg/mL increase)	1.64	0.73–3.70	0.23
Baseline hs-cTnT > 75th percentile	8.05	0.73–88.89	0.089
Baseline hs-cTnT > 14 pg/mL	9.51	0.86–105.19	0.066
Baseline hs-cTnT (per doubling)	1.63	0.53–4.96	0.39
Baseline NT-proBNP (per 50 pg/mL increase)	1.03	0.85–1.25	0.75
Baseline NT-proBNP > 150 pg/mL	3.94	0.36–43.52	0.26
Baseline NT-proBNP (per doubling)	1.16	0.48–2.81	0.75
**Trajectory variables**			
LVEF trajectory (per 5% increase)	0.36	0.20–0.65	0.001
LVEF rate of change (per 1%/month greater change in LVEF trajectory)	0.12	0.01–23.15	0.43
hs-cTnT trajectory (per 5 pg/mL increase)	1.25	0.81–1.94	0.31
hs-cTnT rate of change (per 1 pg/mL/month greater change in hs-cTNT trajectory)	0.64	0.23–1.79	0.40
NT-proBNP trajectory (per 100 pg/mL increase)	1.23	1.07–1.42	0.004
NT-proBNP rate of change (per 5 pg/mL/month greater change in NT-proBNP trajectory)	0.72	0.48–1.08	0.11

Results for the baseline variables are from univariable Fine and Gray competing risk regression models. Results for trajectory variables are from joint models of longitudinal and time-to-event data. (S)HR, (subdistribution) hazard ratio; 95% CI, 95% confidence interval; p, Wald test p-value; LVEF, left ventricular ejection fraction; hs-cTnT, high-sensitivity cardiac troponin T; NT-proBNP, N-terminal pro-brain natriuretic peptide.

### Longitudinal evolution of left ventricular ejection fraction and cardiac biomarkers

**FIGURE 2:**
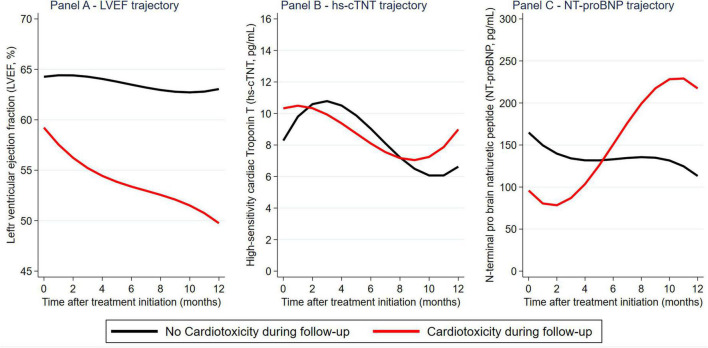
Longitudinal evolution of left ventricular ejection fraction (LVEF), hs-cTnT, and NT-proBNP during trastuzumab-based therapy: Distribution by cardiotoxicity outcomes. Reported graphs are from marginal means predicted at monthly intervals from a mixed effects model.

Within 1 month before and 12 months after the first administration of the HER2-targeted therapy, we analyzed 792, 326, and 326 measurements of LVEF, hs-cTnT, and NT-proBNP. The median number of measurements per patient was 4.3 [range: 1–11] for LVEF, 3.8 [range: 1–9] for hs-cTnT, and 3.8 [range: 1–9] for NT-proBNP. In detail, average LVEF declined by.2%/month in a linear fashion (95%CI: 0.1–0.3, *p* < 0.0001, no evidence for improved fit of quadratic model), whereas hs-cTnT and NT-proBNP showed non-linear changes (Supplementary Figure 5). Patients with and without cardiotoxicity had a strong separation in LVEF trajectory (Figure 2A). In contrast, separation was more modest for NT-proBNP trajectories, and absent for hs-cTnT trajectories (Figures 2B–C).

### Cardiotoxicity risk and longitudinal trajectories of left ventricular ejection fraction, hs-cTnT, and NT-proBNP

The longitudinal trajectory of LVEF harbored prognostic information on cardiotoxicity risk beyond a single baseline measurement. In joint modeling, patients without cardiotoxicity had an average decline in LVEF of.1%/month (95%CI: 0.0–0.3, *p* = 0.017), while the average of those with cardiotoxicity was 1.4%/month (1–1.7, *p* < 0.001) (absolute difference in monthly decline = 1.2%, *p* < 0.001). LVEF trajectory and cardiotoxicity risk were strongly associated. Here, a 1% increase in LVEF at any time of follow-up was associated with a.8-fold relative reduction in the risk of cardiotoxicity (association parameter α = hazard ratio (HR) = 0.81, 95%CI: 0.72–0.92, *p* = 0.001). Importantly, the prognostic association between LVEF trajectory and cardiotoxicity risk prevailed upon multivariable adjustment for baseline LVEF (adjusted association parameter α = 0.79, 95% CI: 0.64–0.97, *p* = 0.028). The rate of change in LVEF (i.e., 1st derivative of association parameter α) was numerically but not statistically significantly associated with cardiotoxicity risk beyond LVEF trajectory (Table 2). In contrast to LVEF, trajectories in cardiac biomarkers were not consistently prognostic for cardiotoxicity risk (although these analyzes were only possible for a subgroup of *n* = 96 patients with available biomarker data). In detail, increasing in hs-cTnT trajectory was not associated with cardiotoxicity risk (Table 2), whereas increase in NT-proBNP trajectory predicted for increased cardiotoxicity (HR for cardiotoxicity per 100 pg/ml increase in NT-proBNP at any time of follow-up = 1.23, *p* = 0.004, Table 2). However, the prognostic association between increasing in NT-proBNP trajectory and cardiotoxicity did not prevail after the multivariable adjustment for baseline NT-proBNP (adjusted HR per 100 pg/ml increase = 0.44, *p* = 0.5), suggesting that NT-proBNP trajectory does not add prognostic information on cardiotoxicity risk beyond a single baseline measurement.

### Personalized prediction of cardiotoxicity risk based on left ventricular ejection fraction trajectories

**FIGURE 3:**
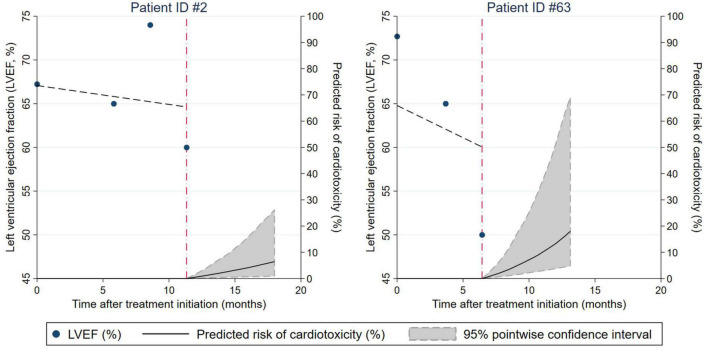
Dynamic prediction of cardiotoxicity risk for individual patients conditional on their prior left ventricular ejection fraction (LVEF) trajectory. Results are from a joint model of longitudinal and time-to-event data. The characteristics of patients ID#2 and ID#63 are described in the Results section, paragraph “Personalized prediction of cardiotoxicity risk based on LVEF trajectories.”

The joint models allowed for a highly personalized, dynamic prediction of cardiotoxicity risk for individual patients conditional on their previous LVEF trajectory. This finding is illustrated according to two sample patients (Figure 3). Patient “ID #2” (left panel) is a 67-year-old lady receiving neoadjuvant chemoimmunotherapy with liposomal doxorubicin, docetaxel, and trastuzumab for triple-positive locally advanced left-sided breast cancer. Throughout the treatment, she had 4 LVEF measurements that were oscillating within the normal range. Conditional on her previous LVEF trajectory, the model predicted a subsequent 6-month cardiotoxicity risk from her last visit at 12 months after a treatment initiation of below 10%. Patient “ID #63” is a 69-year-old lady with triple-positive locally advanced left-sided breast cancer receiving neoadjuvant chemoimmunotherapy with 5-fluorourcil, epirubicin, cyclophosphamide, paclitaxel, and trastuzumab, followed by adjuvant therapy with trastuzumab and letrozole and adjuvant radiotherapy. This patient had declining LVEF during neoadjuvant therapy, and the model predicted a roughly 20% risk of cardiotoxicity for the subsequent 6 months after her last visit. Indeed, she developed asymptomatic cardiotoxicity (LVEF 45% with diffusely impaired contractility) 3 months after starting the adjuvant treatment.

### Risk stratification toward cardiotoxicity based on baseline and dynamic left ventricular ejection fraction cut-offs

**FIGURE 4:**
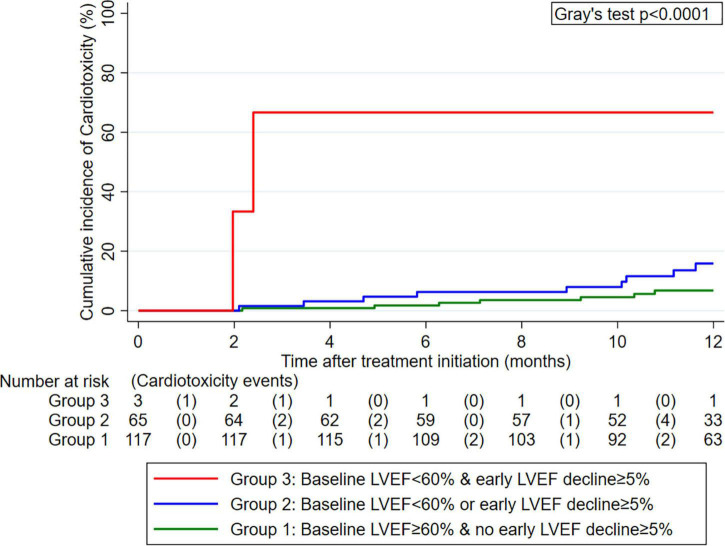
Cumulative 12-month incidence of cardiotoxicity according to three groups defined by pre-treatment left ventricular ejection fraction (LVEF) and early LVEF change after treatment initiation (*n* = 185). Data were estimated with competing risk cumulative incidence estimators, treating death-from-any-cause as the competing event of interest. The numbers below the *x*-axis represent a risk table with the number of patients at risk for cardiotoxicity at the beginning of each interval and the number of patients who developed cardiotoxicity during the pertinent interval in round brackets.

In an exploratory analysis of early LVEF decline, thirty-four patients (18%) developed an LVEF decline of at least 5% from baseline to first follow-up (“early LVEF decline”). Combining the cut-off at 60% for baseline LVEF and 5% for early LVEF decline, one can obtain the following empiric risk stratification: 1-year cardiotoxicity risk was 6.8% in those without early LVEF decline and a baseline LVEF ≥ 60% (*n* = 117, “group 1”), 15.9% in those with an early LVEF decline or a baseline LVEF < 60% (*n* = 65, “group 2”), and 66.7% in those with an early LVEF decline and a baseline LVEF < 60% (*n* = 3, “group 3”) (Gray’s test *p* < 0.001, Figure 4).

### Exploratory analysis: Biomarker-based surveillance in patients at low risk for cardiotoxicity

In the above-defined “group 1” with low cardiotoxicity risk, *n* = 62 patients had a total of 156 hs-cTnT and NT-proBNP readings available after the second LVEF measurement. During the ensuing 12 months, 4 cardiotoxicity events occurred. Within the limitations of these small absolute numbers of patients, readings, and cardiotoxicity events, we did not observe an association between the cardiac biomarker trajectories of the low-risk patients and future cardiotoxicity risk (not shown).

## Discussion

In this study, we examined the utility of LVEF and cardiac biomarkers hs-cTnT and NT-proBNP for dynamic assessment of cardiotoxicity risk in women with HER2+ eBC undergoing trastuzumab-based therapy. Our specific aim was to examine the three variables as a longitudinal trajectory to understand whether monitoring of these markers during therapy informs cardiotoxicity prognosis beyond a single measurement prior to treatment initiation. We found that LVEF harbors strong prognostic information on subsequent cardiotoxicity risk not only as a single baseline measurement but also as a longitudinal trajectory. This allowed for us to develop a joint model for personalized dynamic prediction of cardiotoxicity conditional on each woman’s prior LVEF trajectory. An early decline in LVEF after treatment initiation was particularly prognostic for cardiotoxicity. We could therefore identify a cut-off-based risk stratification rule with baseline LVEF and the first follow-up LVEF. This simple rule could delineate a large subgroup of women who had low cardiotoxicity risk. Regarding hs-cTnT and NT-proBNP, our findings did not support the hypothesis that the two cardiac biomarkers have a strong utility for dynamic cardiotoxicity risk assessment in this setting.

### Interpretation of findings and comparison with the literature: Left ventricular ejection fraction

The association between pre-treatment LVEF and cardiotoxicity risk in the oncologic setting is well-established, and guidelines from leading societies in the field recommend considering periodic LVEF assessment in patients with cancer undergoing cardiotoxic therapy (1, 14). Our study confirms and quantifies the added prognostic information that can be gained from longitudinal LVEF assessment in the oncological important population of women with HER2+ eBC. This can reassure breast oncologists that routine echocardiographic assessment of LVEF prior to treatment initiation and at 3-month intervals during treatment is a meaningful strategy to dynamically identify women with HER2+ eBC at high risk of cardiotoxic complications from trastuzumab-based therapy. As appropriate cut-offs for LVEF and its change are not defined in this setting (1), we derived a risk stratification rule based on pre-treatment LVEF and first follow-up LVEF. This rule could delineate two-thirds of HER2+ eBC patients who have a low risk of cardiotoxicity. We can hypothesize that this large subgroup may be a meaningful population for reducing the intensity of echocardiographic follow-up without compromising cardiac safety. However, a successful prospective and external validation of the risk stratification rule is required before such a hypothesis can be translated to routine clinical care. Moreover, the joint model of longitudinal and time-to-event data allowed for us to obtain highly personalized dynamic cardiotoxicity risk predictions for individual patients based on their LVEF trajectory. It is conceivable that an online implementation of this model could provide breast oncologists and cardio-oncologists with continuously updated cardiotoxicity risk predictions at the point of care, which could then inform the subsequent intervals of echocardiography. Statistical extensions of the joint model to define patient-specific optimal spacing of follow-up intervals based on a longitudinal biomarker trajectory have been developed (15, 16), but again, this would require prospective external validation before clinical implementation in this setting.

### Interpretation of findings and comparison with the literature: Cardiac biomarkers

In contrast to the robust findings on LVEF, the results regarding cardiac biomarkers hs-cTnT and NT-proBNP were mixed. The pre-treatment cardiac biomarkers were correlated poorly with LVEF, elevated pre-treatment hs-cTnT predicted for cardiotoxicity when used with previously described cut-offs (17) but not as a continuous variable, and elevated pre-treatment NT-proBNP showed no association with cardiotoxicity risk. In terms of their longitudinal trajectory, hs-cTnT did not dynamically predict for cardiotoxicity, while a prognostic association between increase in NT-proBNP during treatment and higher cardiotoxicity risk was not independent of baseline NT-proBNP. In a further attempt to study whether longitudinal hs-cTnT or NT-proBNP monitoring may be a valid substitute for echocardiography in two-thirds of the HER2+ eBC patients who were identified as having a low risk of cardiotoxicity by our risk stratification rule, we did not observe the two biomarkers to be associated with cardiotoxicity risk in this subpopulation. Thus, our findings do not provide consistent evidence that pre-treatment cardiac biomarkers hs-cTnT and NT-proBNP or their longitudinal trajectory have a convincing utility for cardiotoxicity risk assessment in HER2+ eBC patients undergoing trastuzumab-based therapy.

Although we may have found stronger cardiotoxicity associations of these biomarkers with a larger sample size, the mixed findings mirror the available literature on this topic. For example, Cardinale et al. and Zardavas et al. found elevated pre-treatment troponins to be predictive of cardiotoxicity in patients with breast cancer undergoing trastuzumab-based therapy, but this finding was confined to patients that had previously received anthracyclines (17–19). Previous anthracycline therapy was not a relevant factor in our cohort of newly diagnosed eBC patients, which could explain the absence of association between hs-cTnT and cardiotoxicity. The literature on NT-proBNP is similarly inconclusive. Feola et al. found elevated pre-treatment BNP in patients with cardiotoxicity (8), while Dodos et al. and Zardavas et al. did not observe such an association (17, 20). In patients with metastatic renal cell carcinoma treated with tyrosine kinase inhibitors, increases in NT-proBNP during treatment were observed, but these increases were not correlated with subsequent cardiotoxic events (21). Increases in BNP subsequent to radiotherapy for left-sided breast cancer were also reported, although their relevance to subsequent cardiotoxicity has not been explored (22).

### Generalizability of study results beyond HER2+ eBC

While trastuzumab-based therapy for breast cancer is the model disease setting for cardiotoxicity in oncology, it needs to be discussed that many studies in the field address biologically and clinically heterogenic spectra of patients across treatment settings and cancer entities, such as patients with and without prior anthracyclines, patients during or after trastuzumab-based therapy or chemotherapy, patients during or after radiotherapy, patients being treated with tyrosine kinase inhibitors for non-breast cancer entities, or patients being treated in the adjuvant, neoadjuvant, or metastatic setting. As the prognostic potential of cardiac biomarkers may strongly differ between, e.g., a cohort of newly diagnosed eBC patients with little comorbidity undergoing adjuvant trastuzumab and a cohort of heavily pretreated patients with metastastic breast cancer on palliative immunochemotherapy, more stringent oncologic delineations of study populations could help in clarifying the utility of cardiac biomarkers for cardiotoxicity risk assessment in oncology.

### Limitations

Finally, several limitations of this study need to be discussed. First, our results apply to women with HER2+ eBC undergoing trastuzumab-based therapy in the curative setting but do not necessarily generalize to other cancer entities or treatment settings. Second, although our cohort underwent a stringent echocardiographic follow-up and was selected in a pre-specified manner from a single academic cancer center, the retrospective nature of the study can be associated with information bias. Third, pre-treatment cardiac biomarkers measurements were only available for a subgroup of our study cohort. This may have led to an underestimation of the association between pre-treatment cardiac biomarkers and cardiotoxicity risk or a limited power to detect true associations between the pre-treatment biomarkers and cardiotoxic outcomes. Fourth, the proposed risk stratification rule must not be implemented in clinical practice before successful external validation. Fifth, consistent with the aim of our study to quantify the utility of LVEF and cardiac biomarker monitoring for eBC patients undergoing trastuzumab-based curative therapy, follow-up was confined to 1 year after treatment initiation. We therefore cannot analyze any long-term cardiotoxic complications occurring years or decades after completion of therapy (23). These complications can be relevant for women with HER2+ eBC who have contemporary cure rates and long-term survival above 80% (3). Fifth, because of the complexity of time-dependent confounding, data on the prescription of relevant co-medications, such as RAAS-blockers, aldosterone antagonists, and beta-blockers, could not be included in the present analysis. This also applied to the potential cardiotoxic effects of concurrent anthracyclines, which were given to a non-negligible proportion of women in this cohort and may have further modified cardiotoxicity risk but also biomarker trajectories. Finally, emerging cardio-oncologic echocardiography techniques, including measurements of global longitudinal strain by speckle tracking or diastolic dysfunction (20, 24–27), were not available.

## Conclusion

In an era of increasing numbers of trastuzumab-based therapies, our results support the routine use of LVEF monitoring as an adequate tool to identify women with HER2+ eBC. We confirm the utility of LVEF for pre-treatment cardiotoxicity risk assessment and define its potential for personalized dynamic risk assessment when used as a longitudinal trajectory during treatment. Cardiotoxicity risk is low in two-thirds of the women with HER2+ eBC who have pre-treatment LVEF ≥ 60% and no early LVEF decline > 5% during trastuzumab-based therapy. In this study, the cardiac biomarkers hs-cTnT and NT-proBNP did not appear to have a convincing utility for cardiotoxicity risk assessment in this setting, either as a single pre-treatment measurement or as a longitudinal trajectory. Future studies should prospectively validate these findings and examine their generalizability to other cancer entities and oncologic treatment settings.

## Data availability statement

The datasets presented in this article are not readily available because public reposition of the data underlying this study is not possible under the current institutional review board decision. However, the data may be shared as part of research cooperation upon reasonable request to the corresponding author. Requests to access the datasets should be directed to PR, peter.rainer@medunigraz.at.

## Ethics statement

This study involved human participants and was reviewed and approved by Ethikkommission der Medizinischen Universität Graz. The patients/participants provided their written informed consent to participate in this study where this was legally required and mandated by the institutional review board mandate (i.e., biobank sampling).

## Author contributions

FP, MP, and PR: idea. FP and PR: conception and design. FP, TG, SF, FM, and EK: data collection. TN, TG, and PR: sample analysis and biobank extraction. FP: statistical analysis and wrote the first draft of the manuscript. PR: revised the first draft of the manuscript. All authors interpreted the data, critically reviewed the manuscript draft and contributed to its writing, agreed with the manuscript’s conclusions and submission in the present form, and authorship criteria according to ICMJE editors met.
