# Supplementary material for: Left ventricular ejection fraction and cardiac biomarkers for dynamic prediction of cardiotoxicity in early breast cancer
Source: Front Cardiovasc Med. 2022 Aug 16;9:933428. doi: 10.3389/fcvm.2022.933428 (PMC9424929; doi:10.3389/fcvm.2022.933428)
Supplement: Supplementary file 1 [file Data_Sheet_1.docx]

Supplementary Material

# Supplementary Figure 1

# Supplementary Figure 2

# Supplementary Figure 3

# Supplementary Figure 4

# Supplementary Figure 5

## Supplementary Figure legends

**Supplementary Figure 1. Cumulative 12-month incidence of cardiotoxicity (n=185).** Data were estimated with a competing risk cumulative incidence estimator, treating death-from-any-cause as the competing event of interest.

**Supplementary Figure 2. Distant Recurrence-free Survival (dRFS) experience of the overall study cohort (n=185).** Results were computed with a Kaplan-Meier estimator.

**Supplementary Figure 3. Cumulative 12-month incidence of cardiotoxicity according to pre-treatment high-sensitivity cardiac Troponin T (hs-cTnT) levels.** Data were estimated with a competing risk cumulative incidence estimator, treating death-from-any-cause as the competing event of interest. Panel A: hs-cTnT cut-off at the 75^th^ percentile of its distribution (Q3, cut-off: 8pg/mL). Panel B: hs-cTnT cut-off at 14pg/mL (representing the established cut-off for the hs-cTnT assay used at our institution).

**Supplementary Figure 4. Cumulative 12-month incidence of cardiotoxicity according to pre-treatment N-terminal pro-brain natriuretic peptide (NT-proBNP) levels.** Data were estimated with a competing risk cumulative incidence estimator, treating death-from-any-cause as the competing event of interest. Cut-off at 150pg/mL (representing the established cut-off for the NT-proBNP assay used at our institution).

**Supplementary Figure 5. Longitudinal evolution of LVEF, hs-cTnT, and NT-proBNP during trastuzumab-based therapy.** Reported curves are derived from linear mixed models (random intercept and random slope) with a quadratic and cubic specification of follow-up time.

## Supplementary Paragraph 1

Among 1,196 echocardiography reports available for our study cohort, LVEF values were documented in 1,076 cases (n=120 LVEF values (10%) missing). However, among the 120 echocardiography reports with missing LVEF values, only 17 reports (1% of all reports) had truly missing LVEF values. Rather, in the remaining reports, LVEF was documented in a semiquantitative manner (e.g. “mildly-reduced systolic function”). To handle these semiquantitative data, we simulated LVEF values in % by randomly drawing from uniform distributions as follows:

| Semiquantitative assessment of LVEF | Random draw from uniform distribution with parameter bound (a,b) |
| --- | --- |
| “normal systolic LV function” | (54,74) |
| “borderline normal systolic LV function” | (54,74) |
| “mildly reduced systolic LV function” | (41,53) |
| “moderately reduced systolic LV function” | (30,40) |
| “moderately to severely reduced systolic LV function” | (20,35) |
